# Are Toll-Like Receptors and Decoy Receptors Involved in the Immunopathogenesis of Systemic Lupus Erythematosus and Lupus-Like Syndromes?

**DOI:** 10.1155/2012/135932

**Published:** 2011-08-16

**Authors:** Giuliana Guggino, Anna Rita Giardina, Francesco Ciccia, Giovanni Triolo, Francesco Dieli, Guido Sireci

**Affiliations:** ^1^Dipartimento di Biopatologia e Biotecnologie Mediche e Forensi, Università degli Studi di Palermo, DIBIMEF, Corso Tukory 211, 90100 Palermo, Italy; ^2^Divisione di Reumatologia, Dipartimento Biomedico di Medicina Interna e Specialistica, Università degli Studi di Palermo, 90133 Palermo, Italy

## Abstract

In this paper we focus our attention on the role of two families of receptors, Toll-like receptors (TLR) and decoy receptors (DcR) involved in the generation of systemic lupus erythematosus (SLE) and lupus-like syndromes in human and mouse models. To date, these molecules were described in several autoimmune disorders such as rheumatoid arthritis, antiphospholipids syndrome, bowel inflammation, and SLE. Here, we summarize the findings of recent investigations on TLR and DcR and their role in the immunopathogenesis of the SLE.

## 1. Introduction

Systemic lupus erythematosus (SLE) and lupus-like syndromes are multiorgan autoimmune diseases whose pathogenesis is multifactorial. Genetic and environmental factors, together with abnormalities of both the innate and the adaptive immune system, are involved. Although faulty activation of autoreactive B lymphocytes and autoantibodies is the hallmark of SLE, other cell subsets are involved in the pathogenesis of the disease. In this context dendritic cells (DC) or other antigen presenting cells (APC) as well as B and T lymphocytes appear to be involved through the activation of autoantibodies, the complement pathway, as well as cytokines and chemokines which in turn activate different effector mechanisms. Recently studies demonstrated an important role of molecular mechanisms of innate immunity in the cascade of events that contribute to the disease [1]. In this paper we focus our attention on the role of two families of receptors such as TLR and DcR involved in the generation of SLE and lupus-like syndromes in human and mouse models. 

## 2. Relevance of TLR in SLE and Lupus-Like Syndromes

Toll-like receptors (TLR) are pattern recognition receptors capable of recognizing specific pathogen-associated molecular patterns (PAMPs) conserved among microorganisms as a part of innate immune system. There are currently twelve known TLR in mammals, which identify common constituents of invading pathogens including double-stranded and single-stranded RNA, unmethylated CpG DNA, bacterial lipopolysaccharide (LPS), lipoproteins, and flagellin [2]. TLRs' binding with several exogenous and/or endogenous ligands activates numerous transcription factors including activating protein-1 (AP-1), nuclear factor-*κ*B (NF-*κ*B), and some of the interferon regulatory factors (IRFs) [3]. A number of adaptor molecules are involved in the TLR signaling pathway, including myeloid differentiation primary response gene 88 (MyD88), TIR domain-containing adaptor protein, TIR domain-containing adapter inducing IFN-*β* (TRIF), and TRIF-related adaptor molecule. TLR are expressed by a great variety of cell types including professional immune cells, for example, DC as well as nonprofessional immune cells, for example, synovial fibroblast-like cells and epithelial cells [4, 5]. Their common theme, however, is to recognize infections and to induce signaling pathways which ultimately lead to the expression of inflammatory mediators and the induction of an immune response. Innate signals were found to play a crucial role in the development of autoimmune nephritis [6]. Given the association between TLR and autoimmunity, a great deal of work has been directed toward understanding how these receptors act in disease progression. Studies have shown two different ways to describe the role of TLR in autoimmune disease. The presence of exogenous antigens, like viral ss-RNA, can stimulate TLR that can activate resident immune cells to initiate and propagate inflammation and autoimmunity, or the TLR can recognize endogenous self-antigens and generate an aggressive autoimmune response in tissues. A crucial point in the generation of autoimmune disease is represented by defective apoptotic cell clearance, common among SLE patients, that can lead to development of antinuclear antibodies [7]. A number of studies have hypothesized that inefficient clearance of apoptotic debris triggers nucleic acid-binding TLR, which induce the B-cell response and subsequent antinuclear antibodies (ANA) production. Studies have demonstrated that as many as one million cells die each second by apoptosis in the human body as a part of normal tissue turnover, although, usually, this phenomenon does not cause autoreactivity [8, 9]. In fact nuclear and cytoplasmic material is sequestered and digested trough autophagy that constitutes an important catabolic mechanism; therefore a failure in autophagy blocks the removal of apoptotic bodies and influences the immunogenicity of death cells [9]. Moreover, if we consider that an apoptotic cell contains a lot of modified self-antigens, the connection between the clearance of apoptotic bodies and autoreactive activation is quite plausible [8, 10, 11]. In 2006 Gaipl et al. demonstrated that an impaired clearance of dying cells may explain accumulation of apoptotic cells in SLE tissues contributing to the development of SLE in humans and mice by several mechanisms [11]. Autoreactive B cells in SLE internalize immune complexes or apoptotic material containing nucleic acids that activate TLRs, causing increased expression of the BAFF receptor TACI and increasing tissue damage mediated by autoantibodies [12–14]. 

Recently, two TLR, namely, TLR7 and 9 have been connected to both human and mouse models of SLE and lupus-like syndromes where they act synergistically with BCR to induce B-cell proliferation [3, 15, 16]. In addition, treatment of lupus-prone mice with a dual inhibitor of TLR-7 and TLR-9 leads to the reduction of autoantibody production and amelioration of disease symptoms [17]. This consideration suggests that aberrant activation of a number of TLR pathways may lead to the initiation and/or perpetuation of SLE. TLR7 and TLR9 blockers, such as antimalarials like hydroxychloroquine, have been used to treat SLE for many years because it can block activation of TLRs by inhibiting endosome maturation. More recently other drugs have been developed to activate or inhibit TLRs [3].

Experiments conducted in two different murine strains have demonstrated that injection of syngeneic late apoptotic thymocytes into wild-type B6 mice led to anti-dsDNA and antihistone antibody production whereas injection into MyD88^−*/*−^ mice had no effect, suggesting that TLR stimulation is important in development of anti-dsDNA antibodies in situations of late apoptotic cell excess. The DNA-binding TLR9 has also been heavily studied in connection with murine lupus in MRL*lpr/lpr *strains. TLR9 deficiency in some lupus models including MRL*lpr/lpr *mice can lead to reductions or alterations in antichromatin antibodies; however, TLR9 deficiency paradoxically leads to disease exacerbation in many experimental models [15]. 

TLR7 has been shown to play an important role in several mouse models of SLE [6, 17–19]. The interaction between TLR7 and ligands directly activates DCs and B cells supporting the expansion of effector T and B lymphocytes specific for autoantigens. In addition, TLR7 activation could be involved in breaking peripheral tolerance mediated by Tregs, important to generate protection against pathogenic autoreactive immunity [18]. Furthermore, TLR7 activation by exogenous and endogenous TLR7 ligands impairs Treg generation and function. Hackl et al. demonstrated that TLR ligands have differential effects on Treg generation. TLR7 and similarly TLR9 ligands but not TLR4 ligand (e.g., LPS) reduced de novo generation of Tregs from naïve T cells. This effect appears to be mediated by IL-6 with a minor role for IFN-*γ* and IL-4 in inhibiting Treg generation in the presence of TLR7 ligand, which is in accordance with a recent report describing the influence of Th1-/Th2-polarizing cytokines on Treg differentiation [20]. IL-6 inhibits conversion of naïve T cells into Tregs and supports Th17 differentiation [21–23]. Expression of ROR*γτ* and IL-17 mRNA in FOXP3^+^ T cells generated in the presence of TLR7 ligand suggested the possibility of a Th17 regulatory cells [24]. Tregs were originally believed to be a stable Th-cell lineage but since then, several studies have clearly shown that Foxp3 expression can be downregulated in subpopulations of natural as well as induced Tregs allowing conversion into Th1, Th2, or Th17 effector cells under the influence of polarizing cytokines *in vitro *and in inflammatory environments *in vivo* [24]. In summary, TLR7-modified immunoregulation by Tregs contributes to the breakdown of peripheral tolerance and development of autoimmunity in SLE, where activation of TLR7 by endogenous ligands was shown to play a role in the pathogenesis. New therapeutic approaches for SLE and lupus-like syndromes would be better directed on modifying Foxp3 expression by interfering with TLR7 activation or by blocking downstream effector cytokines such as IL-6.

TLR4 is another TLR thought to be involved in the pathogenesis of glomerulonephritis in SLE. LPS-mediated immune responses are mediated through Toll-like receptor 4 (TLR4) on the target cells. Overexpression of TLR4 in mice has led to autoimmune glomerulonephritis and lupus-like disease [25], suggesting that LPS-mediated TLR4 signaling plays a pivotal role in lupus nephritis [26]. TLR4 as well as the commensal flora has been shown to be essential for the production of anti-dsDNA and the immune complex-mediated glomerulonephritis in transgenic mice expressing surface gp96 [25]. Lee et al. demonstrated that the activations of immature APC and autoreactive B cells in anti-dsDNA Tg mice by TLR4 signaling correlated with an increase in production of IFN-*γ*, IL-10, and anti-dsDNA production. Therefore, discovery and development of novel TLR4 and LPS antagonists may be a new paradigm for the therapy of SLE [26].

Various studies concerning autoimmune diseases in lupus-prone mouse models and from genome-wide association studies in human lupus patients and *in vitro *studies with cells obtained from patients showed a correlation between nucleic acid-binding TLR and the progression and severity of lupus and other autoimmune diseases [27]. Although there has not been a new drug approved for the treatment of lupus, current investigation regarding the targeting of TLRs and their downstream effectors is promising and therefore merits further investigation. The accumulation of evidence pointing towards the involvement of TLRs in autoimmunity has opened the door for potential therapeutic interventions directed to modulate Toll-like receptors expression and their signaling pathways [15]. 

## 3. Decoy Receptor Role in Humans and Murine SLE-Like Models

Decoy receptors are ‘‘silent scavengers” of CC chemokines and cytokines, which play a key role in damping inflammation and tissue damage [28]. Decoy receptors recognize certain inflammatory cytokines with high affinity and specificity, but are structurally incapable of signaling or presenting the agonist to signaling receptor complexes. They act as a molecular trap for the agonist and for signaling receptor components [29]. Inhibition of T cell death could modify clonal shrinkage after clonal expansion during an immune response, resulting in the survival of abnormal self cross-reactive T cells and, potentially, autoimmune disease. 

At present, the DcR6 receptor, expressed on endothelial cells, hematopoietic stem cells (HSC), megakaryocytes, mast cells, and DC, is the focus of intense investigation in the field of inflammation since it binds many inflammatory CC chemokines (CCL2, CCL3, CCL4, CCL5, CCL7, CCL8, CCL11, CCL14, CCL22, and weakly CCL17), without triggering any signals in target cells [28]. Interestingly, although previous studies have always focused on the anti-inflammatory role of DcR6 in relationship with its capacity to scavenge circulating proinflammatory CC chemokines, recent studies in experimental autoimmune encephalomyelitis (EAE), a mouse model of multiple sclerosis, have suggested that DcR6 functions may be different and can change depending on the setting of the inflammatory conditions. In EAE models, DcR6-deficient mice showed a significantly lower immune response with a reduced inflammatory leukocyte infiltration in the spinal cord and, consequently, a decreased demyelization [28].

Recent studies have focused their attention on another decoy receptor: DcR3. It is a tumor necrosis factor receptor family member and is a secreted protein that can enhance cell survival by interfering with multiple apoptosis pathways. It is a decoy receptor for the Fas ligand (FasL) and can inhibit FasL-induced apoptosis. 

 DcR3 seems to function like the soluble Fas (sFas) in terms of binding FasL and competing with membrane Fas. Hyperproduction of DcR3 may be involved in the acquisition of autoimmunity (DcR3 and T-cell activation). In 2007 Han et al. demonstrated that DcR3 overexpression could lead to a lupus-like syndrome [30]. Since then, several reports have identified DcR3 as a possible parameter and risk factor for SLE and that its elevated levels in serum can contribute to enhancing T cells activation in SLE. SLE patients showed significantly high serum level of DcR3, and the mean serum DcR3 level was higher for those with active disease (SLE disease activity index (SLEDAI) > 10) compared with that in patients with inactive disease (SLEDAI < 10). In addition soluble DcR3-Fc enhanced T-cell proliferation and increased interleukin-2 (IL-2) and IFN-*γ* production via co-stimulation of T cells in response to cell death. Moreover, enhanced T-cell reactivity to DcR3-induced costimulation was demonstrated in lymphocytes from patients with SLE, suggesting that the elevated serum DcR3 may be associated with enhanced T-cell activation *in vivo*. It is likely that the DcR3-Fc-induced T-cell proliferation is via interaction between DcR3 and LIGHT (the cognate receptor). Previous studies have demonstrated that DcR3 could bind to LIGHT and transducer costimulatory signals into both human and murine T cells [31]. It is still not clear whether there is any particular T-cell subset responding to DcR3-induced costimulation, and what effector function will develop after triggering by DcR3.

Normal T cells express low levels of DcR3 [32], and healthy individuals have near-background serum levels [33]. DcR3 expression is augmented in activated T cells [32], thus probably represent a fine-tuning mechanism to balance the need for clonal expansion and subsequent massive activation-induced cell death of T cells. A dysfunction of this balance due to failed AICD might lead to pathologic consequences, such as autoimmune disease including SLE. Studies conducted with DcR3 transgenic mice with actin promoter-driven expression of human DcR3 demonstrated these mice to manifest a lupus-like syndrome after 5-6 months of age, thus, suggesting that DcR could play a role in the complex intertwinement of mechanism that drive the pathogenesis of lupus-like syndromes. The hypothetical role of decoy receptors in lupus-like syndromes could be different to that reported in experimental Mtb infection [34]; in systemic autoimmune disease the increase of decoy receptors, binding more chemokines, could amplify the recruitment of activated cells (neutrophils, macrophages, lymphocytes, dendritic cells, etc.) increasing the inflammatory state of the tissue damaged by autoimmune reactions. 

## 4. Concluding Remarks

**Figure 1:**
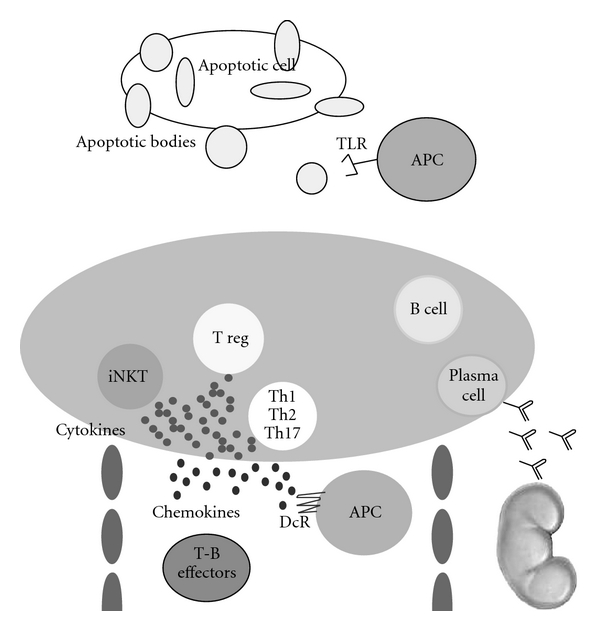
Mechanisms involved in the pathogenesis of SLE and lupus-like syndromes. Different cells and receptors contribute to the activation of B cells secreting autoantibodies; these Immunoglobulins cause tissue damage.

The pathogenesis of SLE and lupus-like syndromes in humans and mice is multifactorial. Several factors can contribute to the immunopathogenesis of SLE and lupus-like syndromes (sex, age, hormones, infectious background, environmental factors, drugs, abnormalities of both innate and adaptive immune system). Here, we draw your attention to two kinds of receptors associated with innate immunity as players in the pathogenesis of SLE and lupus-like syndromes: TLRs (7, 9, and 4) as possible receptors of autoantigenic molecules and decoy receptors as molecules able to propagate autoimmune inflammation acting in synergy to cause autoimmune inflammations (Figure 1). Our paper, highlighting the new players in this autoimmune disorder, could offer the opportunity to design new immunotherapeutical approaches for systemic autoimmune diseases.
